# Evaluating movement disorders in pediatric patients receiving risperidone: a comparison of spontaneous reports and research criteria for TD

**DOI:** 10.1186/1753-2000-1-3

**Published:** 2007-06-26

**Authors:** Gahan J Pandina, Cynthia A Bossie, Young Zhu, Georges M Gharabawi

**Affiliations:** 1 Ortho-McNeil Janssen Scientific Affairs, L.L.C., Titusville, NJ, USA; 2 Ortho-McNeil Janssen Scientific Affairs, L.L.C., Titusville, NJ, USA; 3 Current address: Roche Pharmaceuticals, Nutley, NJ, USA

## Abstract

**Background:**

Movement disorders (MD) in children are relatively common and may be associated with medication use. Objective methods (ie rating scales) and specific research criteria may be helpful in identifying MD-related adverse events that would otherwise not be apparent from spontaneous reports. We assessed whether more stringent and rigorous criteria would provide MD rates similar to those derived subjectively from spontaneous reports.

**Methods:**

MDs were assessed in children with disruptive behavior disorders (DBDs) and subaverage intelligence receiving risperidone. Data were from three 1-year, open-label studies in subjects 4–14 years old. Dyskinesia severity was rated by the Extrapyramidal Symptom Rating Scale (ESRS) dyskinesia subscale. Tardive dyskinesia (TD) was defined: mild dyskinesia (scores 2, 3) in two anatomical areas; or moderate dyskinesia (score ≥ 4) in one area for ≥ 4 weeks in subjects without dyskinesia at baseline (scores 0, 1).

**Results:**

The mean (± SD) age of subjects was 9.4 ± 2.4 years, the mean (± SD) risperidone dose was 1.6 ± 0.7 mg/day, and the mean (± SD) exposure was 317.8 ± 104.5 days. ESRS data were available for 668 subjects. Mean ESRS scores were low throughout the study. At baseline, 655 subjects had no dyskinetic symptoms. One subject met predefined TD criteria after a risperidone dose reduction. Symptoms persisted for 4 weeks, resolving with continued treatment and no dosage change. Two different subjects had TD by spontaneous adverse-event reports, with dyskinetic symptoms at 1–2 visits, and symptoms that resolved after treatment discontinuation. Thirteen subjects had dyskinesia at baseline; their mean ESRS dyskinesia scores decreased at endpoint.

**Conclusion:**

Using objective rating scales and research criteria, low-dose risperidone was associated with low risk of TD and other MDs in children with DBDs in three large 1-year studies. Careful, objective evaluation of emergent MDs during all stages of treatment is essential for identifying treatment-emergent TD.

## Background

Disorders that affect movement in children are relatively common and may be inherited or acquired [1]. Noniatrogenic movement disorders (MDs) can include dystonia, dyskinesias, chorea/ballismus, myoclonus, tics, tremor, stereotypies, and parkinsonism [1,2]. These can be difficult to distinguish from each other, and some (eg, tics) are found in association with comorbid conditions such as attention-deficit/hyperactivity disorder (ADHD), obsessive-compulsive disorder (OCD), anxiety disorders, mood disorders, learning disorders, sleep disorders, conduct and oppositional behavior, and self-injurious behavior [2]. MDs may also be drug induced; medications that induce movement disorders include antipsychotics, antiepileptics, beta-adrenergic agonists, amphetamines, and lithium. The identification and classification of MDs generally, and drug-induced movement disorders specifically, is quite complex. Although subjective methods (ie spontaneous adverse events or observations) have traditionally been used to determine MD rates, objective research instruments and defined criteria may be more sensitive than subjective approaches.

Among the best-characterized drug-induced movement disorders are those associated with antipsychotic treatment [2]. Antipsychotic agents are used in children and adolescents to treat a range of psychiatric and neurologic disorders, including schizophrenia, disruptive behavior disorders (DBDs), Tourette's syndrome, and autism spectrum disorders [3-7]. However, while it is acknowledged that antipsychotics have a definite role in the treatment of pediatric subjects, there is a dearth of well-controlled efficacy and safety data in this population [3].

Among antipsychotics of any class, the atypical antipsychotic risperidone is the best studied in children and adolescents. Several large, well-controlled studies have examined the efficacy of risperidone in children with DBDs and subaverage intelligence (Table 1). Two double-blind, placebo-controlled, short-term (six-week) studies noted significant improvements on the primary outcome measure, the conduct problem subscale of the Nisonger Child Behavior Rating Form (NCBRF) [8,9]. Long-term studies of up to two years' duration have indicated that early improvements in behavioral symptoms are sustained over time and are associated with improvements in cognitive functioning consistent with age-appropriate gains [4,10-15]. An eight-week, double-blind, placebo-controlled study in 101 children with autistic disorder found that risperidone was significantly superior to placebo (*P *< 0.001) in reducing tantrums, aggression, or self-injurious behavior [16]. Positive responses to risperidone at eight weeks were maintained at six months in two thirds of the children [17]. An eight-week, double-blind, placebo-controlled study in 34 subjects (26 of whom were children) evaluated the efficacy of risperidone for Tourette's syndrome. Risperidone significantly reduced tic severity in comparison with placebo among pediatric subjects [5].

**Table 1:** Short-Term and Long-Term Studies of Risperidone in Pediatric Subjects With Disruptive Behavior Disorders (DBDs)

**Citation**	**Population **	**Dosing**	**Duration**	**Results**
Aman et al 2002 [8]	118 children aged 5–12 with DBDs and subaverage IQ	0.02–0.06 mg/kg/day RIS or PBO	6 weeks	Significant improvements over PBO by week 1 on the NCBRF conduct problem subscale; significant improvement over PBO on all other NCBRF subscales
Snyder et al 2002 [9]	110 children aged 5–12 with DBDs and subaverage IQ	0.02–0.06 mg/kg/day RIS or PBO	6 weeks	Significant improvements over PBO by week 1 on the NCBRF conduct problem subscale; significant improvement over PBO on all other NCBRF subscales
Findling et al 2004 [11]	107 children aged 5–14 with DBDs and subaverage IQ previously participating in a 6-week DB study	0.02–0.06 mg/kg/day RIS (mean dose, 1.64 mg/day)	1-year OL extension	Significant improvements on the NCBRF conduct problem subscale, most notably during the first 4 weeks; significant change from baseline on all other NCBRF subscales
Turgay et al 2002 [15]	77 children aged 5–12 with DBDs and subaverage previously participating in a 6-week DB study	0.02–0.06 mg/kg/day RIS (mean dose, 2.38 mg/day)	48-week OL extension	Significant improvements on the NCBRF conduct problem subscale in subjects previously receiving PBO in DB study; improvements were maintained in subjects previously treated with risperidone during DB study
Croonenberghs et al 2005 [10]	504 children aged 5–14 years with DBDs and subaverage IQ	0.02–0.06 mg/kg/day RIS (mean dose, 1.6 ± 0.03 mg/day)	1 year	Significant improvement on the NCBRF conduct problem subscale over baseline as early as week 1; improvements were maintained over the course of the study
Reyes et al 2006 [13]	48 children from [10] aged 7 to 15 with DBDs, subaverage IQ, and comorbid ADHD	0.02–0.06 mg/kg/day (mean dose, 1.83 mg/day)	12-month OL extension of Findling et al	Significant improvements on the NCBRF conduct problem subscale were maintained through the second year of treatment
Reyes et al 2006 [14]	35 children from [10] aged 5–15 years with DBDs, subaverage IQ, and comorbid ADHD	0.02–0.06 mg/kg/day (mean dose, 1.92 mg/day)	24-month OL extension of Findling et al	Symptoms continued to be well controlled, as measured by CGI

IQ indicates intelligence quotient; RIS, risperidone; PBO, placebo; NCBRF, Nisonger Child Behavior Rating Form; DB, double-blind; OL, open-label; ADHD, attention-deficit/hyperactivity disorder; CGI, Clinical Global Impressions.

While studies of risperidone have to date suggested treatment benefits, clinical decision making regarding the use of any antipsychotic agent in younger patients must include an assessment of the potential risk for movement disorders. Overall, risperidone treatment in children with DBDs, autistic disorder, or Tourette's syndrome was shown to be well tolerated, with low ratings of movement disorder severity and few movement disorder adverse events [4,5,8-11,13-16]. Even so, treatment-emergent tardive dyskinesia (TD), because of its persistence and potential to worsen in severity, remains a particular concern. In adult subjects, atypical antipsychotics are associated with a lower risk for TD than are conventional agents and have been suggested to demonstrate antidyskinetic properties in subjects with preexisting TD [18]. In a recent meta-analysis, atypical antipsychotics were associated with a lower mean annual incidence of TD (0.8%) than was haloperidol (5.4%) [19]. No long-term studies have evaluated antipsychotic-associated movement disorders in children and adolescents. Such information is critical in this potentially vulnerable population, particularly when long-term treatment may be required. Given the rising use of atypical antipsychotics in pediatric populations across an expanding range of disorders and specialties, it may be beneficial to apply objective research critiera to determine whether they are more sensitive in identifying movement disorders related to atypical antipsychotic use than are spontaneous reports or observations.

This report is the first to assess TD by defined research criteria [20,21] in a large population of children and adolescent subjects receiving an atypical antipsychotic. Data were derived from three one-year, open-label, long-term studies of risperidone in children with DBDs and subaverage intelligence [10,11,15].

## Methods

Data were from two one-year, open-label extension studies of short-term, placebo-controlled studies [8,9], and a one-year, open-label study in children with DBDs and subaverage intelligence. Detailed descriptions of patient populations, study designs, treatment, measures, and data analyses have been published previously [10,11,15]. Institutional review boards at participating sites approved individual studies. Written informed consent was provided by each study participant (if capable) and by the guardian or legal representative. A responsible party was required to accompany the participant during study visits, to provide reliable assessments, and to dispense study medications.

### Subjects

Participants were recruited from the clinical practices of the investigators and colleagues; local school districts; self-referrals via newsletter stories; and newspaper and radio advertising. Subjects were screened by parent rating on various instruments (eg, NCBRF [22], Aberrant Behavior Checklist (ABC) [23]), followed by a physical and psychiatric history, and clinician examination. Subjects were included if they had a DSM-IV diagnosis [20] of conduct disorder (CD), oppositional defiant disorder, or DBD not otherwise specified (DBD-NOS) [20,24]; a rating of ≥ 24 on the conduct problem subscales of the NCBRF; a DSM-IV Axis II diagnosis of mild or moderate mental retardation [20] or borderline intellectual functioning with an IQ of ≥ 36 and ≤ 84; and a Vineland Adaptive Behavior Scale score ≤ 84 [25]. Subjects had to be healthy, and aged between 4 and 12 years (extension studies) or between 4 and 14 years (separate open-label study). Exclusion criteria included a diagnosis of pervasive developmental disorder, schizophrenia, or other psychotic disorder; head injury as a cause of intellectual disability; a seizure disorder requiring medication; females who were sexually active and without reliable contraception; serious or progressive illness or clinically abnormal laboratory values; a history of TD, neuroleptic malignant syndrome, or hypersensitivity to any antipsychotic drug; and known presence of human immunodeficiency virus. The open-label extension studies required that participants had completed at least two weeks of treatment in the preceding double-blind study and met criteria for continuation in the study. Subjects were excluded if > 3 weeks had elapsed since their participation in the previous double-blind trial, or if they had experienced a hypersensitivity reaction to trial medication, extrapyramidal symptoms not controlled by medication, an adverse event possibly related to risperidone, or an adverse event for which they were withdrawn from the previous trial.

### Treatment

Subjects who participated in the open-label extension studies received a daily risperidone dose of 0.02 to 0.06 mg/kg, with dosing initiated and established in the double-blind studies [8,9]. The separate one-year, open-label study included a three-day screening period and single-blind treatment with placebo for one week to rule out placebo responders, followed by entry into the trial by the remaining subjects. Treatment with risperidone was initiated in the morning or afternoon, beginning with 0.01 mg/kg for the first two days and changing to 0.02 mg/kg on day 3. The dosage could be increased weekly thereafter by 0.02 mg/kg/day to a maximum of 0.06 mg/kg/day. Allowed concomitant medications included those for preexisting medical conditions, psychostimulants (could be continued for comorbid ADHD for those on a stable dose for at least 30 days prior to entry), sleep medication (antihistamines, chloral hydrate, and melatonin), and anticholinergic medication for any extrapyramidal symptoms arising during the study.

### Measures

Efficacy and safety assessments were completed and have been detailed elsewhere [8-11,15]. Movement disorders were assessed using the Extrapyramidal Symptom Rating Scale (ESRS) [26] at baseline and at weeks 1, 2, 3, 4, 8, 12, 16, 20, 24, 36, 48, and endpoint. Dyskinesia was measured with the ESRS seven-item dyskinetic movement subscale (subcale items 51–57). These items evaluate lingual, jaw, buccolabial, truncal, choreoathetoid movements (upper and lower extremities), and other involuntary movements. Each item is rated from 0 (absent) to 6 (severe and constant). Raters were trained on the ESRS using training tapes at a multicenter investigators' meeting held to standardize procedures. Investigators and/or designated raters performed ESRS ratings of video-recorded interviews of patients. Videotapes were available at study sites to improve the performance of raters and to monitor inter-rater reliability. Initiation of a study at any site required evidence of inter-rater ESRS reliability and certification of raters. Inter-rater reliability required that ≥ 80% of item ratings of the complete scale should be ± 1 point of expert ratings, and that ≥ 70% of ratings on individual items of each ESRS subscale should be ± 1 point of expert ratings.

In this post hoc analysis, criteria for treatment-emergent TD were consistent with Schooler and Kane [21] and DSM-IV [20]. These criteria require that the subject has dyskinetic movements of at least mild severity in two or more anatomical areas or of moderate severity in one or more areas for a duration of ≥ 4 weeks; has onset of symptoms beyond week 4 of discontinuing an oral antipsychotic or beyond week 8 of discontinuing a depot antipsychotic; and has no other conditions that could cause movement disorders. Since adequate information on prior antipsychotic use was not available for this population, for the purposes of this analysis, it was assumed that these patients were neuroleptic naïve. As a consequence of this conservative approach, any dyskinesias in patients at the beginning of the study were not considered to be withdrawal dyskinesias.

ESRS criteria for dyskinesia were two or more scores of 2 or 3 (mild), or one score of ≥4 (moderate or greater severity) on the ESRS dyskinesia subscale. TD was defined as dyskinesia at two or more consecutive visits (covering four weeks' duration) in subjects without dyskinetic symptoms at baseline (all seven ESRS dyskinesia items equal 0 or 1). ESRS score assignments of mild as a rating of 2 or 3 and moderate as a rating of 4 on the physician's examination for dyskinesia subscale were based on a prior analysis [27].

### Data analysis

Movement disorders were evaluated in all patients from the initiation of risperidone treatment, regardless of study phase. This represented the beginning of the double-blind phase in patients who participated in either of the double-blind studies and who had been randomized to receive active treatment. This approach enabled the analysis to include patients who had an onset of dyskinetic symptoms during the six-week double-blind exposure period. For all other patients, movement disorders were evaluated from the initiation of risperidone treatment at the beginning of the open-label extensions. Data were combined from the studies. Analyses included all subjects who had a baseline ESRS assessment and at least two scheduled post-baseline ESRS assessments. Changes in scores from baseline to endpoint (last observation carried forward) were analyzed using two-sided paired *T *tests or repeated measure analysis. Mean values and their standard deviation are provided as descriptive statistics.

## Results

Baseline and postbaseline ESRS data were available for 668 subjects. The majority of subjects (484, 72.5%) were from the 1-year, open-label study [10]. The mean age ± standard deviation (SD) of subjects was 9.4 ± 2.4 years, and the mean (± SD) IQ was 64.9 ± 13.4. The majority of subjects were male (81.9%) and most were white (79.8%). The major AXIS I diagnoses were CD (n = 285, 42.7%), alone (n = 139) or in combination with ADHD (n = 146); and ODD (n = 280, 41.9%), alone (n = 117) or in combination with ADHD (n = 163). A total of 268 (40.2%) subjects were diagnosed with borderline intellectual functioning, 273 (40.9%) with mild mental retardation, and 126 (18.9%) with moderate mental retardation. Subjects were excluded if they were receiving antipsychotics immediately prior to entry into the open-label study by Croonenberghs and colleagues or the double-blind, placebo-controlled studies that preceded the open-label extensions. The mean (± SD) dose of risperidone in all studies combined was 1.6 ± 0.7 mg/day, and the mean (± SD) exposure was 317.8 ± 104.5 days. Twenty-six percent of patients received stimulant medications during the trial. A total of 472 (70.7%) of the 668 subjects completed the respective studies.

### Movement disorders

Mean ESRS scores for the total patient population were low throughout the study (Table 2). Significant decreases from baseline to endpoint were noted for the subjective overall rating (items 1–11; *P *= 0.0002, *df *= 667, *T *= -3.73) and the physician's examinations for akathisia (item 28; *P *< 0.0001, *df *= 667, *T *= -5.72). One hundred fifty-two patients (22.8%) reported a movement disorder-related adverse event during the study. Among the 50 patients who discontinued prematurely owing to adverse events, 13 were reported to have a movement disorder-related adverse event during the study. Seven of 13 patients who discontinued due to a movement disorder-related adverse event reported one or more movement disorders at the time of discontinuation. In five of 13 patients, movement disorders were the only reported adverse event at discontinuation (case 1, dyksinesia; case 2 dyskinesia and tardive dyskinesia, case 3, tardive dyskinesia; case 4, extrapyramidal disorder, hypertonia, hypokinesia; case 5, extrapyramidal disorder). One patient had dyskinesia at study entry. Twenty-nine patients (4.3%) received antiparkinsonian agents during the study. There was no significant difference in mean dyskinesia scores between patients with or without stimulant use at baseline or endpoint (baseline, *P *= 0.763; endpoint, *P *= 0.198).

**Table 2:** Movement Disorder Ratings in the Total Study Population

**ESRS Subscale or Item**	**Possible Range of Scores**	**Mean Baseline Score (± SD)**	**Mean Endpoint Score (± SD)**	*P *Value for Change From Baseline* (*df*, test value)
Subjective overall rating (items 1–11)	0–33	0.80 ± 1.59	0.60 ± 1.39	0.0002 (667, *T *= -3.73)
Physician's examination for parkinsonism (items 13–30)	0–108	1.07 ± 3.18	0.88 ± 2.35	0.0596 (667, *T *= -1.89)
Physician's examination for akathisia (item 28)	0–6	0.41 ± 1.07	0.19 ± 0.70	< 0.0001 (667, *T *= -5.72)
Physician's examination for dyskinesia (items 51–57)	0–42	0.17 ± 1.02	0.12 ± 0.73	0.2155 (617, *T *= -1.24)
CGI of severity of parkinsonism (item 59)	0–8	0.08 ± 0.46	0.11 ± 0.45	0.2331 (667, *T *= 1.19)
CGI of severity of dystonia (item 60)	0–8	0.05 ± 0.37	0.04 ± 0.31	0.7534 (667, *T *= -0.31)
CGI of severity of dyskinesia (item 58)	0–8	0.06 ± 0.39	0.08 ± 0.44	0.3535 (667, *T *= 0.93)

ESRS indicates Extrapyramidal Symptom Rating Scale; SD, standard deviation; CGI, Clinical Global Impressions.

*Two-sided *P *value for paired *T *test.

### Assessment of emergent tardive dyskinesia

At baseline, 655 subjects (98.1%) were rated as being without dyskinetic symptoms (all ESRS dyskinesia item scores 0 or 1). During the study, one (0.2%) patient met the objective criteria for TD (severity and duration of symptoms). This patient had a score of 1 on three of the seven dyskinesia items at baseline. The dyskinetic movements meeting the TD criteria emerged at week 16 after a second reduction in risperidone dose (at weeks 8 and 12), suggesting that this was a withdrawal dyskinesia (Table 3). Symptoms persisted to week 20, for a total duration of four weeks, and resolved by the next visit with continued treatment of a stable, reduced dose of risperidone. This patient did not receive anticholinergic medication and completed the 48-week study period.

**Table 3:** Characteristics in the One Subject With Treatment-Emergent Tardive Dyskinesia as Per Defined Research Criteria

Gender	Male	
Age	10 y	
Diagnosis	Oppositional defiant disorder	
Intellligence quotient (IQ)	59	
NCBRF total score		
Baseline	33	
Endpoint	31	
Time point	Risperidone dose (mg/day)	Dyskinesia score*
Baseline	0.000	3
Week 1	0.343	3
Week 2	1.200	3
Week 3	1.814	3
Week 4	1.900	3
Week 8	1.784	---
Week 12	1.300	4
Week 16	1.300	7
Week 20	1.300	7
Week 24	1.300	1
Week 36	1.300	2
Week 48	1.300	0

NCBRF indicates Nisonger Child Behavior Rating Form.

*Extrapyramidal Symptom Rating Scale (ESRS) physician's examination for dyskinesia, items E51–57.

### Spontaneous adverse events reports of TD

Two subjects (exclusive of the two subjects described above) who did not meet the ESRS criteria for TD were reported to have TD as a spontaneously reported adverse event. These two subjects were originally reported in the one-year study by Croonenberghs et al [10]. Table 4 provides the subjects' characteristics, NCBRF total scores, risperidone doses, and dyskinesia scores. The first patient was reported to have abnormal movements at week 48 (final study visit). The investigator rated the event as severe and very likely related to study medication. No anticholinergic medication was administered, and the subject received no additional doses of risperidone. The patient was improved at a follow-up visit 10 days later and recovered completely in approximately two months. The second patient reportedly exhibited occasional movements of the lips after 133 days of risperidone treatment. The investigator rated this event as mild and very likely related to study medication and reduced the risperidone dose from 1.6 to 1.0 mg/day. Seven days later, the patient displayed marked buccal labial movements reported as moderate TD. Risperidone treatment was discontinued at that time; he recovered without further treatment in approximately two weeks.

**Table 4:** Patient Characteristics in the Two Subjects With Tardive Dyskinesia Reported as an Adverse Event

	**Case 1**		**Case 2**	
Gender	Female		Male	
Age	9 y		7 y	
Diagnosis	Attention-deficit/hyperactivity disorder – oppositional defiant disorder		Disruptive behavior disorder	
Intelligence quotient (IQ)	40		52	
NCBRF total score				
Baseline	47		42	
Endpoint	11		31	

Timepoint	Risperidone dose (mg/day)	Dyskinesia score	Risperidone dose (mg/day)	Dyskinesia score
Baseline	0.300	0	0.300	0
Week 1	0.667	0	0.778	0
Week 2	0.914	0	1.000	0
Week 3	1.114	0	1.000	1
Week 4	1.200	0	1.000	0
Week 8	1.090	0	1.540	0
Week 12	1.000	0	1.600	0
Week 16	1.000	3	1.580	0
Week 20	1.000	2	0.300	0
Week 24	0.813	1		
Week 36	0.800	1		
Week 48	0.783	9		

NCBRF indicates Nisonger Child Behavior Rating Form.

### Effect of treatment on subjects with existing dyskinesia

Thirteen subjects (2.0%) had dyskinetic symptoms at baseline. The mean age (± SD) of these subjects was 8.5 ± 1.8 years, and 69% were male. The mean IQ (± SD) was 63.4 ± 12.3. Twelve subjects were white, and one was black. The mean (± SD) risperidone dose was 1.5 ± 0.6 mg/day, and the mean (± SD) exposure was 325.8 ± 104.4 days. Two of the 13 subjects discontinued the study, both for adverse events. In one patient, the reason for discontinuation was a movement disorder. There were no obvious differences between these subjects and the total population with respect to clinical symptoms, IQ, diagnosis, sex, or age. Eleven subjects were from the separate one-year, open-label study [10], and two were from the study of Findling and colleagues [11]. Two subjects received anticholinergics during the study period, and two were taking stimulants.

Mean ESRS scores at baseline and endpoint are provided in Table 5. Overall scores were higher for these subjects with dyskinetic movements than for those not having dyskinetic symptoms at baseline. Mean severity of movement disorder symptoms declined at endpoint for all measures, significantly so for the physician's examination for parkinsonism, akathisia, and dyskinesia, and for the Clinical Global Impressions (CGI) for parkinsonism and dyskinesia (all *P *< 0.05).

**Table 5:** Extrapyramidal Symptom Rating Scale Scores in the 13 Subjects With Dyskinesia at Baseline

**ESRS Subscale or Item**	**Possible Range of Scores**	**Mean Baseline Score (± SD)**	**Mean Endpoint Score (± SD)**	*P *Value for Change From Baseline* (*df*, test value)
Subjective overall rating (items 1–11)	0–33	4.38 ± 3.28	3.00 ± 4.02	0.2277 (12, *T *= -1.27)
Physician's examination for parkinsonism (items 13–30)	0–108	9.85 ± 8.21	3.23 ± 3.19	0.0161 (12, *T *= -2.80)
Physician's examination for akathisia (item 28)	0–6	2.00 ± 1.58	0.69 ± 0.95	0.0083 (12, *T *= -3.16)
Physician's examination for dyskinesia (items 51–57)	0–42	5.46 ± 3.60	2.23 ± 3.17	0.0166 (12, *T *= -2.78)
CGI of severity of parkinsonism (item 59)	0–8	1.08 ± 1.44	0.15 ± 0.38	0.0395 (12, *T *= -2.31)
CGI of severity of dystonia (item 60)	0–8	0.92 ± 1.50	0.08 ± 0.28	0.0591 (12, *T *= -2.09)
CGI of severity of dyskinesia (item 58)	0–8	2.00 ± 1.22	0.85 ± 1.41	0.0119 (12,*T *= -2.96)

ESRS indicates Extrapyramidal Symptom Rating Scale; SD, standard deviation; CGI, Clinical Global Impressions.

*Two-sided *P *value for paired *T *test.

## Discussion

Risperidone has been shown to be efficacious in children with DBDs and subaverage IQ [4,8-11,13-15]. Emerging evidence suggests that it also may be efficacious in children with autism and other neurologic disorders [6,16]. The benefits of antipsychotic treatment in pediatric patients, however, must be carefully weighed against the risks. The risk of movement disorders is one such important aspect to consider, particularly when choosing among antipsychotic drugs. This analysis represents the first assessment of TD by defined research criteria in children and adolescents receiving an atypical antipsychotic. In three long-term trials that included 668 subjects, low-dose risperidone treatment in pediatric subjects with DBDs and subaverage IQ was associated with a low risk of movement disorders, including akathisia. These data are consistent with a newly published study of low-dose risperidone in pediatric patients [28]. One patient met the defined research criteria for TD, which emerged after a dosage reduction. It persisted for four weeks and resolved with continued treatment and no dosage change. No patient was identified with persistent TD beyond 4 weeks' duration.

Notably, there was a disparity between the case of dyskinesia, which persisted for 4 weeks and was identified by defined research criteria for TD, and the two TD cases identified by spontaneous adverse event reports. These cases were mutually exclusive. Neither case identified by adverse event reporting met the research criteria for treatment-emergent TD. This may be due, in part, to the fact that raters in the trial are trained to use the ESRS, but clinicians were not instructed to use a standardized diagnosis of TD for adverse event reporting. Furthermore, the collection of adverse events via spontaneous reporting by patients or caregivers may be limited because of a lack of awareness of dyskinetic movements. A similar finding – that cases of emergent TD identified by defined research criteria and those identified by spontaneous event reporting are mutually exclusive – was noted in a study of another database [29]. Nonetheless, the low rate identified via spontaneous reporting is not less sensitive than the formal research criteria, as both methods revealed similar rates.

Thirteen subjects had dyskinetic symptoms at baseline. The mean severity of dyskinesia symptoms in these subjects decreased significantly when they were treated with risperidone. The physician's examination of ESRS showed an overall reduction of reversible movement disorders during the study, particularly parkinsonism and akathisia, as well as dyskinetic movements. Of note, these 13 patients also had higher mean ESRS scores for parkinsonism. It may be difficult to distinguish between drug-induced and spontaneous movement disorders (parkinsonism, akathisia, etc) and some symptoms of illness, such as repetitive behaviors and hyperkinesias. It is also unclear whether patients with cognitive impairment are more susceptible to neurologic side effects. This difficulty may have been a factor in certain cases, despite training in research practices related to movement disorders. A more systematic evaluation of prior antipsychotic use and assessment of spontaneous dyskinetic movement would provide a better understanding of these 13 subjects. The presence of dyskinetic symptoms in children and adolescents with neurodevelopmental or psychotic disorders before initiation of risperidone treatment was also noted in a retrospective chart review reported by Demb and Nguyen. Seven of 36 children had positive ratings on one or more items of the Dyskinesia Identification System: Condensed User Scale before treatment was initiated [30]. Further, a study that investigated abnormal involuntary movements in 390 antipsychotic-naive children and adolescents in foster care found that 4.1% of subjects had at least 2 ratings of 2 (mild) or 1 rating of 3 (moderate) on any of the first 7 items on the AIMS. The prevalence of movement disorders by these criteria was significantly higher in subjects with lower intelligence (IQ ≤69; 10.6%) compared with those who were more intellectually competent (IQ ≥70; 2.1%) [31]. It appears that lower intelligence itself may confer a risk for movement disorders, and may help explain the presence of dyskinesia at baseline in the 13 subjects.

## Limitations

Limitations of this report include the open-label, noncomparative study design, which precluded comparisons with other antipsychotic agents, either conventional or atypical. Since these studies were not designed to measure emergent TD, limited historical data were available regarding prior medication use that could impact patients' susceptibility to drug-induced movement disorders.

Although these studies were not designed to assess TD, the large patient numbers, the frequency of the ESRS evaluations, and the long duration of these studies provided an opportunity to better understand this pressing clinical concern. An additional strength of this report was the use of the ESRS, a comprehensive scale for the assessment of movement disorders that provides specificity in the detection of dyskinesias separate from other movement disorders, such as dystonias.

Infrequent visits for the assessment of TD limited the ability to assess the persistence of dyskinesia in patients with an onset of symptoms after week 24. Further, TD that would have emerged beyond the study period described here would also be undetected. Two subsets of patients from the study by Croonenberghs et al [10] were followed for an additional one year (n = 48) (21) or two years (n = 35) of risperidone treatment [13]. Although subjects were not evaluated for treatment-emergent TD using the defined criteria applied in this analysis, EPS were rarely reported as an adverse events. There were no reports of TD [13,28].

## Conclusion

It is essential to carefully assess movement disorders and TD, and to distinguish those that are treatment-emergent from those that may be behavioral characteristics of some pediatric disorders. This analysis of three large, long-term trials highlights the need for careful, objective evaluation of emergent movement disorders during all stages of treatment. These data further suggest that treatment with low-dose risperidone in pediatric subjects with DBDs is associated with a low rate of TD and other movement disorders. This safety information, coupled with efficacy results in other psychiatric and neurologic disorders, is essential for clinical decision making in young patients, particularly when long-term use of antipsychotics is anticipated. Additional large, rigorous studies examining the benefits and risks of antipsychotics in children and adolescents are needed.

## Competing interests

Drs. Pandina, Bossie, and Zhu are employees of Ortho-McNeil Janssen Scientific Affairs, L.L.C., Titusville, NJ. At the time of study, Dr. Gharabawi was also an employee of Ortho-McNeil Janssen Scientific Affairs, L.L.C., Titusville, NJ.
